# Assessment of Pain Complaints and Perioperative and Delayed Complications of Hysteroscopy Performed Under Local Anesthesia—A Retrospective Analysis

**DOI:** 10.3390/jcm14165646

**Published:** 2025-08-09

**Authors:** Agnieszka Lach, Maciej Wilczak, Adam Malinger, Adrian Nowak, Piotr Piekarski, Adrian Mruczyński, Kinga Bednarek, Karolina Chmaj-Wierzchowska

**Affiliations:** Department of Maternal and Child Health and Minimally Invasive Surgery, Poznan University of Medical Sciences, 60-535 Poznan, Poland; mwil@ump.edu.pl (M.W.); adam.malinger@ump.edu.pl (A.M.); anowak@ump.edu.pl (A.N.); piotr.piekarski@ump.edu.pl (P.P.); adrianmrucz@gmail.com (A.M.); kinga.bednarek88@gmail.com (K.B.)

**Keywords:** mini-hysteroscopy, complications, pain

## Abstract

Modern, small-diameter endoscopic instruments, such as resectoscopes (e.g., the GUBBINI System) and mini-hysteroscopes, are widely used in clinical practice. These tools allow endoscopic procedures to be conducted without cervical dilation, often in an outpatient setting, and under local anesthesia alone. **Background/Objectives**: The present retrospective study aimed to analyze the perioperative and delayed complications of hysteroscopy performed under local anesthesia. This study also assessed the pain experienced during hysteroscopy under local anesthesia, depending on the type of procedure performed. **Methods**: A retrospective analysis was conducted in 1945 patients who underwent hysteroscopy under local anesthesia at the Center for Hysteroscopy, Heliodor Święcicki Gynecological and Obstetrical Clinical Hospital, Karol Marcinkowski Medical University, Poznań, Poland, between January 2021 and December 2023. Hysteroscopic procedures were performed with the GUBBINI Mini Hystero-Resectoscope through a paracervical block using lignocaine. **Results**: The procedure was discontinued in 46 patients, accounting for 2.36% of all hysteroscopies. The most common reasons for procedure discontinuation were severe pain and uterine perforation, accounting for 52.8% and 13% of discontinued procedures, respectively. The complication rates were low: uterine perforation occurred in 0.3% of cases (*n* = 6), and late complications requiring readmission occurred in 0.2% (*n* = 3). The average pain intensity score for all the patients was 2 points (2.8 ± 2.14). **Conclusions**: Our study confirmed that hysteroscopy performed under local anesthesia is a safe and effective diagnostic and therapeutic method for selected uterine pathologies, noting increased risks in cases such as extensive intrauterine adhesions. The low complication rates in both the perioperative and postoperative stages indicate the high safety profile of this procedure, particularly when performed by experienced personnel using standardized, validated protocols.

## 1. Introduction

Hysteroscopy, an endoscopic procedure, is currently considered the gold standard for diagnosing and treating pathologies of the uterine cavity and cervical canal in both premenopausal and postmenopausal women [1,2]. Because of its minimally invasive nature, hysteroscopy enables direct visualization of the interior anatomy of the uterine cavity and simultaneous treatment of the detected lesions, increasingly replacing traditional uterine curettage procedures that require general anesthesia [3]. Modern, small-diameter endoscopic instruments, such as resectoscopes (e.g., the GUBBINI System) and mini-hysteroscopes, are widely used in clinical practice. These tools allow endoscopic procedures to be conducted without cervical dilation, often in an outpatient setting and under local anesthesia alone [4,5]. This approach provides several benefits to patients, including a reduced risk of anesthetic complications, lower cost, shorter hospital stay, and rapid return to daily activities [6,7,8,9].

However, not all hysteroscopic procedures can be performed under local anesthesia alone. In cases of extensive pathological changes or substantial pain, deeper anesthesia—including general anesthesia—may be required [10,11,12]. Advances in hysteroscopic technology and techniques have remarkably increased diagnostic efficacy, enabling the precise identification of abnormalities such as endometrial polyps, submucosal fibroids, intrauterine adhesions, retained products of conception, displaced intrauterine devices (IUDs), adenomyosis, or congenital uterine anomalies [13]. The most common indications for diagnostic hysteroscopy include abnormal uterine bleeding, infertility, and recurrent pregnancy loss [14,15,16]. Therapeutic procedures involving diagnostic hysteroscopy include polypectomy [17], the removal of submucosal fibroids [18], endometrial ablation [19], IUD extraction, and the treatment of intrauterine adhesions [20,21].

Mild, moderate, and severe pain after outpatient hysteroscopy have been reported in approximately 66%, 22%, and 12% of patients, respectively [22]. Moreover, the pain intensity may depend on the type of procedure, operator experience, duration, presence of pathological changes, and individual sensitivity to pain [1]. Although several studies have reported the use of hysteroscopy under local anesthesia, comprehensive data on the complication rates associated with this method remain limited. The widespread application of ultrasound-based diagnosis has resulted in increasing detection of intrauterine pathologies, necessitating the development of effective, safe, and minimally invasive treatment techniques [13]. Although hysteroscopy is a relatively safe procedure, both early (perioperative) and late complications can occur. The most common early complications include uterine perforation (0.13% for diagnostic hysteroscopy [DH] and 0.5–3% for operative hysteroscopy [OH]), bleeding, and absorption of uterine distension media. The incidence of fluid overload syndrome (operative hysteroscopy intravascular absorption syndrome) due to excessive absorption of distension fluid is very low—less than 1%. Late complications may include intrauterine adhesions and pelvic infections, with post-hysteroscopy infection rates not exceeding 1%, which does not justify the use of routine prophylactic antibiotics [1]. Hysteroscopy is currently a critical diagnostic modality in modern gynecology for diagnosing and treating intrauterine pathologies. Given its increasing importance, it is essential to develop strategies to prevent and effectively manage hysteroscopy-related complications.

Hence, the present retrospective study aimed to analyze the perioperative and delayed complications of mini-hysteroscopy performed under local anesthesia. This study also assessed the pain experienced during hysteroscopy under local anesthesia depending on the type of procedure performed—OH or DH.

## 2. Materials and Methods

A retrospective analysis was conducted on 1945 patients who underwent hysteroscopy under local anesthesia at the Center for Hysteroscopy, Heliodor Święcicki Gynecological and Obstetrical Clinical Hospital, Karol Marcinkowski Medical University, Poznań, Poland, between January 2021 and December 2023. All procedures were performed in an outpatient hysteroscopic setting via paracervical block with lignocaine.

For analytical purposes, the patients were assigned to two groups according to the type of hysteroscopic procedure performed, reported symptoms, and ultrasound findings:

- Group I included 835 women who underwent DH with endometrial biopsy, typically as part of a preoperative evaluation for a planned laparoscopic supracervical hysterectomy, an infertility assessment, recurrent miscarriage, or abnormal uterine bleeding without a detectable intrauterine pathology.
- Group II included 1110 women who underwent OH for the removal of endometrial polyps or submucosal fibroids confirmed via ultrasound.

Patients were considered eligible to undergo hysteroscopy under local anesthesia with lignocaine according to the following criteria: the detection of abnormal uterine bleeding or the presence of intrauterine abnormalities such as endometrial polyps, submucosal fibroids, or endometrial hyperplasia. The study included women in the first phase of their menstrual cycle or in the postmenopausal period. Final eligibility was based on the findings of gynecological and ultrasound examinations. Patients with heavy vaginal bleeding (>100 mL/24 h) or vaginal or cervical infections were excluded from the study. Patients with heavy vaginal bleeding were excluded due to the risk of limited intrauterine visibility, which could compromise safety and procedural completeness. This exclusion criterion also helped to reduce potential bias in pain perception. Each patient underwent a thorough medical history-taking, including their age, body weight, height, history of gynecological surgery, allergies, number of deliveries and miscarriages, previous cervical or uterine procedures, and general health status. Each patient first underwent a gynecological and ultrasound examination and then provided written informed consent for the procedure.

Approximately 30 min before the planned procedure, all patients received 100 mg of intravenous ketoprofen. Their vital signs, including their heart rate, blood pressure, oxygen saturation, and respiratory rate, were monitored during the procedure. Local anesthesia was administered approximately 10 min before the hysteroscope was inserted. Two paracervical injections (at 4 and 8 o’clock positions), each containing 10 mL of 1% lignocaine solution, were administered using a Hystero-Block needle.

All procedures were performed by operators with comparable experience levels, each with at least 5 years of training in operative hysteroscopy. All were certified in the use of the GUBBINI system and had performed a minimum of 150 outpatient hysteroscopies prior to the study period. The patients were positioned on a standard gynecological table. The uterine cavity was distended using an isotonic saline solution (0.9% NaCl) in a continuous flow system, with the intrauterine pressure maintained at 120 mmHg. The procedure was conducted using the GUBBINI System Mini Hystero-Resectoscope. The patients remained conscious throughout the procedure and could observe a real-time video feed of the procedure on a monitor.

The patients were asked to report the maximum pain intensity they experienced during the procedure, immediately after its completion and before leaving the treatment room. Pain intensity was assessed using the visual analog scale (VAS) score, ranging from 0 (no pain) to 10 (worst imaginable pain). According to the scores, pain was classified as mild (0–3), moderate (4–7), or severe (8–10) [17]. Prior to the procedure, the patients received an explanation of the scale and were provided with guidelines to rate their pain intensity on the scale. One limitation of this study is its single-center design and execution by highly experienced hysteroscopists, which may limit the generalizability of our findings to settings with fewer resources or lesser procedural volume. Moreover, although pain was assessed immediately after the procedure using a standardized VAS, the use of postprocedural pain reporting may introduce recall bias. Psychological factors such as preoperative anxiety and the brief delay between the procedure and pain rating may have influenced the patients’ perceptions. Future studies should consider real-time or intraoperative pain monitoring and the inclusion of less specialized centers to enhance external validity.

The data analysis was conducted using Statistica (Cloud Software Group, Inc., Fort Lauderdale, FL, USA, 2023, Data Science Workbench, version 14) and Microsoft Excel (Microsoft Office, Redmond, WA, USA, 2019, version 2205). The Mann–Whitney *U* test and Kruskal–Wallis ANOVA were used for group comparisons. Kendall’s tau and chi-square maximum likelihood (Chi^2^) tests were applied to assess correlations between variables. A *p*-value of <0.05 was considered statistically significant.

## 3. Results

### 3.1. Characteristics of the Study Group

The hysteroscopies were generally brief procedures, with most lasting between 10 and 25 min. The mean age of the patients was 45.75 ± 12.69 years (median: 44 years; range: 19 to 87 years). The mean body mass index (BMI) was 25.62 ± 5.42 kg/m^2^ (median: 24.38), with a mean height of 165.75 ± 6.19 cm (median: 165 cm) and a mean body weight of 70.36 ± 15.26 kg (median: 67 kg). Age and body mass parameters are presented in Table 1.

### 3.2. Procedure

The procedure was discontinued in 46 patients, accounting for 2.36% of all hysteroscopies. A retrospective analysis of medical records revealed that the procedure was discontinued significantly more often in DH cases than in OH cases. Age and BMI showed no correlation with procedure discontinuation (Table 2).

The odds ratio for procedure discontinuation in the diagnostic group compared to the operative group was OR = 3.36 (95% CI: 1.75–6.45), indicating a significantly increased risk of discontinuation in the diagnostic group.

### 3.3. Pain Intensity Level

The pain intensity level experienced during the hysteroscopy procedure ranged from 0 to 10 points. The average pain intensity score for all the patients was 2 points (2.8 ± 2.14). Table 3 presents the distribution of pain intensity levels according to VAS scores across the entire study group.

#### 3.3.1. Correlation Between Pain Intensity Level and Age in the Study Group

**Figure 1:**
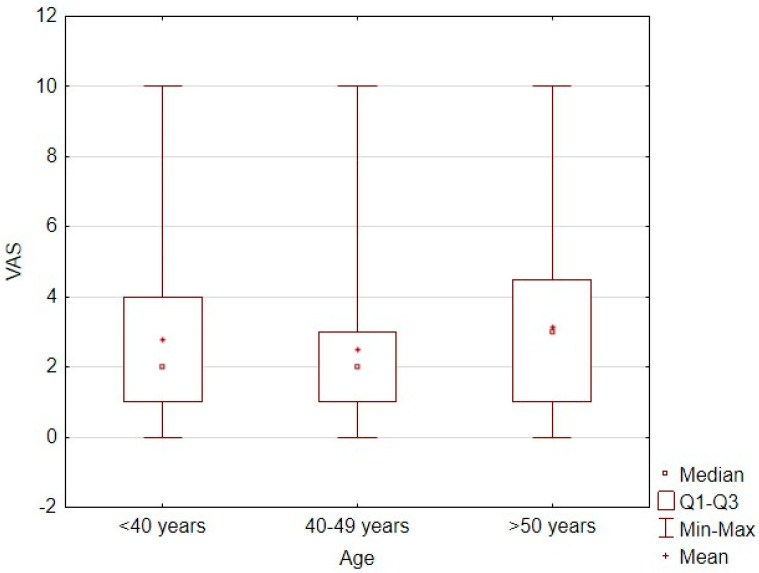
Correlation plot between age and pain intensity level measured by VAS score.

Patients aged 40–49 years had significantly lower pain intensity levels than did those under 40 years of age (z = 2.45, *p* = 0.043) and those over 50 years of age (z = 4.8, *p* < 0.001; Table 4). However, age showed no significant linear correlation with the pain intensity level measured on the VAS (τ = 0.03, *p* = 0.057; Figure 1).

#### 3.3.2. Correlation Between Pain Intensity Level and BMI

**Figure 2:**
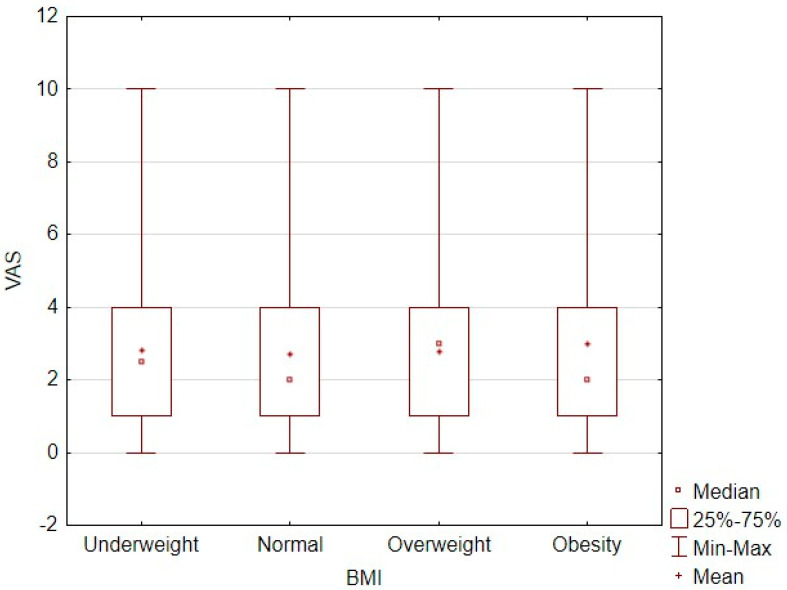
Correlation plot between BMI and pain intensity level measured by VAS score.

No significant differences were observed in pain intensity levels according to the patients’ BMI. Additionally, BMI and VAS scores showed no significant linear correlation (τ = 0.002; *p* = 0.31; Figure 2). The correlation between the pain intensity level and body weight category is shown in Table 5.

#### 3.3.3. Correlation Between Pain Intensity Level and Type of Hysteroscopic Procedure

No significant differences in pain intensity levels were observed between patients who underwent DH or OH (Table 6).

#### 3.3.4. High-Pain Subgroup Analysis

Among all patients, 375 (19.3%) reported moderate-to-severe pain (VAS ≥ 4). A logistic regression analysis was performed to identify predictors of high pain. The model included age, BMI, procedure type (diagnostic vs. operative), and presence of intrauterine adhesions.

None of the analyzed variables reached statistical significance. Although the presence of adhesions showed a trend toward an increased risk of higher pain (OR = 1.38; 95% CI: 0.85–2.23), this association did not reach significance (*p* = 0.193).

#### 3.3.5. Linear Regression Model

An analysis of variance for the assumed predictors indicated that age category and withdrawal from the procedure were statistically significant predictors. The average VAS value was 0.32937 higher for individuals under 40 years of age and 0.56067 higher for those over 50 years of age than for those aged 40–49 (when other variables were controlled for). Individuals who withdrew from the procedure had an average VAS value 1.92745 higher than that of individuals who did not withdraw. Other predictors, such as age, height, BMI, body mass index, and hysteroscopy type, did not show a significant effect on the VAS in this model. The coefficient of determination R^2^ indicates that approximately 3.53% of the variance in the VAS is explained by the independent variables in the model. This indicates that the model explains a very small portion of the total variance. The overall F-test was statistically significant (*p* < 0.001), indicating that at least one of the independent variables significantly contributes to the prediction of the dependent variable. Despite the low R^2^, the model as a whole is statistically significant.

### 3.4. Reasons for Procedure Discontinuation

The most common reasons for procedure discontinuation were severe pain and uterine perforation, accounting for 52.8% and 13% of discontinued procedures, respectively. The reasons for procedure discontinuation are shown in Table 7. In cases classified as “risk of uterine perforation,” the hysteroscopy was discontinued due to strong intraoperative resistance, anatomical uncertainty, or suspected cervical canal abnormality. These were preventive decisions made by experienced operators to avoid iatrogenic injury in the absence of direct visualization of the uterine cavity.

### 3.5. Effect Sizes

A very small effect size confirmed that the type of hysteroscopy had a negligible effect on pain levels. Because the significance level of the test was above 0.05, the difference between the groups was not statistically significant. A moderate effect size indicated that discontinuation of the procedure was associated with significantly higher pain levels (*p* < 0.05). Table 8 presents the effect sizes depending on the type of hysteroscopy and the need to abandon the procedure.

A very small effect size indicated that age had little effect on pain levels. Because the significance level was below 0.05, the difference between the groups was statistically significant. A negligible effect size confirmed that body weight did not significantly affect pain levels. Because the significance level was above 0.05, the difference between the groups was not statistically significant. Table 9 presents the effect sizes depending on the age and BMI vs. the VAS.

A low effect size confirmed the lack of a relationship between age and withdrawal from surgery. As the significance level of the test was above 0.05, no significant relationship was found between the groups. A low effect size confirmed the lack of a relationship between body weight and withdrawal from the procedure. Since the significance level of the test was above 0.05, no significant relationship was found between the groups. Table 10 presents the effect sizes depending on age and BMI vs. the need to abandon the procedure.

The effect size for the relationship between forgoing the procedure and the type of hysteroscopy was low, indicating a small effect. Because the significance level of the test was below 0.05, a statistically significant relationship was found: the type of hysteroscopy was associated with the decision to forgo the procedure. Based on the odds ratio, we estimated that the odds of forgoing the procedure were more than three times higher for one type of hysteroscopy. Table 11 presents the effect size depending on the type of hysteroscopy vs. the need to abandon the procedure.

### 3.6. Early Perioperative Complications

Uterine cavity perforation occurred in six patients following the hysteroscopy procedure. After a thorough case analysis, the main cause of iatrogenic uterine injury was found to be adhesions located within the cervical canal and uterine cavity. All patients were admitted to the ward for general condition monitoring and extended observation. A control ultrasound examination was performed on the following day, and the patients were subsequently discharged home in good general condition with recommendations for oral antibiotic therapy. Table 12 shows details of the individual clinical cases.

### 3.7. Late Complications

Three patients were readmitted urgently within 8 weeks after the hysteroscopy procedure. Table 13 presents the occurrence of late complications that required rehospitalization and treatment.

## 4. Discussion

Our study of almost 2000 hysteroscopy cases performed in an outpatient setting under local anesthesia demonstrated the high effectiveness and safety of this procedure in terms of both the diagnosis and treatment of pathological changes within the uterine cavity. The low rate of complications encountered in this study—including perforations, bleeding, and infections—is at the lower end of the range reported in the literature, thus confirming the validity of the adopted protocols and the high competence of the medical team.

In particular, the uterine perforation rate observed in our study (0.3%) is at the lower end of the range reported in the literature for operative hysteroscopy. In a prospective multicenter study, Jansen et al. (2000) reported an overall perforation rate of 0.76%, with significantly higher risk during operative procedures compared to diagnostic ones [23]. Similarly, Aas-Eng et al. (2017) reported perforation and complication rates ranging from 0.5% to 1.5%, depending on the surgical complexity and underlying uterine pathology [17].

Vitale et al. (2022), in a cross-sectional international survey on in-office hysteroscopy, emphasized that the rates of complications such as perforation are influenced by the device type, operator experience, procedure setting (inpatient vs. outpatient), and anesthesia protocol [20]. Their findings highlighted that when standardized protocols are followed, office-based hysteroscopy can be both safe and effective.

These observations are consistent with the existing literature, which indicates that uterine perforation—while one of the most common complications of hysteroscopy—is typically self-limited and does not result in serious outcomes when promptly recognized and managed. In most cases, conservative management is sufficient, and surgical intervention is not required unless there is evidence of visceral or vascular injury [24].

Clinically significant bleeding during operative hysteroscopy is uncommon, with an estimated incidence of approximately 0.61% [17]. Such bleeding is often associated with uterine perforation but may also result from cervical trauma, bleeding at the resection site, or underlying bleeding diathesis. In our study, bleeding events requiring intervention were rare, reflecting both the precision of the technique and appropriate patient selection. When bleeding occurred, it was effectively managed intraoperatively using bipolar coagulation loops and antifibrinolytic agents, tailored to the distension medium employed. In cases of postoperative bleeding, additional measures such as uterotonics or balloon tamponade (e.g., Foley catheter) were available, ensuring comprehensive hemorrhage control.

Although no formal infection monitoring protocol was implemented in this study, the observed postoperative infection rate was very low. This likely reflects a combination of strictly aseptic technique, appropriate patient selection, and the short duration of procedures. Previous studies have reported that the incidence of inflammatory complications following hysteroscopic procedures is below 1%, supporting the notion that routine antibiotic prophylaxis is not required [25,26]. Our findings are in line with this, as postoperative infections were rare and effectively minimized through rigorous preoperative screening and attentive postoperative care.

Procedure discontinuation, mainly due to severe pain or anatomical difficulties, occurred more frequently in patients who underwent DH. This might be due to the limited scope of the procedure and the absence of sedation. The use of intravenous premedication, paracervical anesthesia, and small-diameter hysteroscopes substantially increased pain tolerance, which aligned with the findings of Bettocchi et al. [27].

While the overall mean VAS score was low, nearly one in five patients (19.3%) experienced moderate-to-severe pain (VAS ≥ 4). This subgroup warrants particular attention, as it may reflect insufficient analgesic control despite local anesthesia and premedication. However, a multivariate regression analysis did not identify any significant predictors among age, BMI, procedure type, or intrauterine adhesions. These findings suggest that individual pain perception and psychological factors may play a substantial role, highlighting the need for individualized analgesic protocols and further research on non-biological predictors.

From an organizational perspective, hysteroscopy under local anesthesia provides several benefits, such as no requirement for hospitalization, a short procedure time, and the rapid resumption of daily activities by the patient. These characteristics of outpatient hysteroscopy are the key advantages of this procedure, particularly in the context of the increasing demand for minimally invasive procedures in healthcare. Similarly, in our present study, the procedure duration was short, and the patients could rapidly resume their full professional activity.

Our results were also influenced by psychosocial factors. Patients aged 40–49 years reported the lowest pain intensity level during hysteroscopy when compared to both younger (<40) and older (>50) women. These results are consistent with those of some previous studies, indicating that age may influence pain perception during gynecological procedures. Troia et al. (2019) found that patients over 50 years of age reported greater discomfort, possibly related to atrophic changes in the vaginal mucosa and cervical canal occurring after menopause [28]. While patients aged 40–49 years reported the lowest pain scores, these differences may be multifactorial and warrant further prospective investigation. Despite these differences in age groups, age and VAS pain scores showed no significant linear correlation. This finding suggests that age alone may not be a direct determinant of pain perception but, rather, interacts with other variables such as hormonal status, anxiety, health experience, or procedure type.

Our results also showed no significant differences in pain intensity levels according to the subjective body weight category; moreover, no significant linear correlation was found between the BMI and the pain intensity level. Previous retrospective studies, such as that by Nowak et al. (2023), also reported similar conclusions, wherein the BMI did not significantly influence the pain intensity during hysteroscopy [29]. This finding also aligns with that of Rodriguez et al., who analyzed a large sample of outpatient hysteroscopy patients and observed no significant relationship between BMI and pain severity [30]. Thus, BMI alone does not appear to be a predictor of pain severity in these procedures. Although our findings may suggest some associations, they do not allow definitive conclusions regarding predictive pain modeling. This hypothesis could be explored in future studies.

Our findings emphasize the importance of individualized preprocedural care, particularly in addressing pain-related anxiety, providing clear patient education, and selecting appropriate premedication. In addition, factors such as patient age and the quality of interaction between the patient and physician should be taken into account during treatment planning. While patient–provider communication was not directly assessed in this study, previous research suggests that it may significantly affect perceived comfort during the procedure. Finally, our results support the use of local anesthesia to successfully replace general anesthesia in most routine cases, offering benefits such as reduced hospital stays, lower costs, and fewer anesthesia-related risks.

In summary, this study conducted at our center demonstrated that hysteroscopy under local anesthesia meets all the requirements of modern gynecological procedures: it is safe, effective, cost-efficient, and focused on patient needs. These results justify the implementation of this treatment modality as a standard approach in selected clinical groups, particularly given the increasing demand for same-day procedures.

## 5. Conclusions

Our study confirmed that diagnostic and operative hysteroscopy performed under local anesthesia with the GUBBINI system is a safe and effective approach for managing a wide range of intrauterine pathologies in the outpatient setting. However, caution is advised in patients with extensive intrauterine adhesions or distorted uterine anatomy, where the risk of complications such as perforation may be higher. The overall low rate of perioperative and postoperative complications highlights the strong safety profile of this technique, particularly when conducted by experienced operators using standardized and validated protocols.

## Figures and Tables

**Table 1:** Anthropometric measurements of the study patients.

		*n* = 1945	%
Age	under 20 years	5	0.26%
	21–30 years	171	8.79%
	31–40 years	541	27.81%
	41–50 years	698	35.87%
	51–60 years	262	13.47%
	61–70 years	170	8.74%
	71–80 years	80	4.11%
	81–90 years	23	0.93%
BMI	Underweight—BMI < 18.5 kg/m^2^	58	2.99%
	Normal body weight—BMI: 18.5–24.9 kg/m^2^	1020	52.50%
	Overweight—BMI: 25–29.9 kg/m^2^	508	26.15%
	Class 1 obesity—BMI: 30–34.9 kg/m^2^	226	11.63%
	Class 2 obesity—BMI: 35–39.9 kg/m^2^	94	4.84%
	Class 3 obesity—BMI ≥ 40 kg/m^2^	37	1.9%

**Table 2:** Procedure discontinuation.

		Procedure Discontinuation		*χ* ^2^	*p*
		Yes	No		
Age	<40 years	12 (1.85%)	635 (98.15%)	2.13	0.35
	40–49 years	16 (2.21%)	708 (97.79%)		
	>50 years	18 (3.11%)	561 (96.89%)		
BMI	Underweight	0 (0%)	58 (100%)	3.57	0.31
	Normal	22 (2.16%)	998 (97.84%)		
	Overweight	14 (2.76%)	494 (97.24%)		
	Obesity	10 (2.8%)	347 (97.2%)		
Hysteroscopy	Diagnostic	32 (3.83%)	803 (96.17%)	14.94	<0.001
	Operative	13 (1.17%)	1097 (98.83%)		

**Table 3:** Distribution of pain intensity levels in the study group according to VAS scores.

VAS Scores (0–10 Points)	*n* = 1945	%
0	243	12.49
1	312	16.04
2	459	23.60
3	376	19.33
4	197	10.13
5	146	7.51
6	66	3.39
7	54	2.78
8	57	2.93
9	25	1.29
10	11	0.57

**Table 4:** Age and pain intensity levels assessed via the VAS.

Age	n	Mean ± SD	Min–Max	Me [Q1–Q3]	*H*	*p*
<40 years	647	2.8 ± 2.12	0–10	2 [1–4]	23.77	<0.001
40–49 years	723	2.51 ± 1.95	0–10	2 [1–3]		
>50 years	576	3.15 ± 2.33	0–10	3 [1–4.5]		

**Table 5:** Correlation between BMI and pain intensity level measured by VAS score.

BMI	*n*	Mean ± SD	Min–Max	Me [Q1–Q3]	H	*p*
Underweight	58	2.81 ± 2.18	0–10	2.5 [1–4]	1.997	0.57
Normal	1018	2.72 ± 2.11	0–10	2 [1–4]		
Overweight	507	2.77 ± 2.05	0–10	3 [1–4]		
Obesity	356	2.99 ± 2.33	0–10	2 [1–4]		

**Table 6:** Association between type of hysteroscopic procedure and pain intensity level measured by VAS score.

Hysteroscopy Type	*n*	M ± SD	Min–Max	Me [Q1–Q3]	U	*p*
Operative	1110	2.82 ± 2.11	0–10	2 [1–4]	451,604.5	0.41
Diagnostic	835	2.77 ± 2.18	0–10	2 [1–4]		

**Table 7:** Reasons for procedure discontinuation.

Reasons for Procedure Discontinuation	*n* = 46	%
Severe pain	24	52.8%
Risk of uterine perforation	2	3.8%
Uterine perforation	6	13%
Extensive intrauterine adhesions	1	1.9%
Large number of lesions within the uterine cavity	1	1.9%
Decrease in heart rate—bradycardia	1	1.9%
High blood pressure values	2	3.8%
No pathological changes in the uterine cavity	1	1.9%
Maximum use of fluid medium	3	5.7%
Cervical canal occlusion	5	11.3%

“Risk of uterine perforation” refers to preventive discontinuation based on intraoperative tactile resistance or a suspicion of uterine wall thinning or abnormal angulation.

**Table 8:** Effect sizes depending on the type of hysteroscopy and the need to abandon the procedure.

	U Mann–Whitney		
Hysteroscopy	Statistics	*p*	Effect size
VAS	451,605	0.412	0.0214
Procedure abandonment	Statistics	*p*	Effect size
VAS	29,543	<0.001	0.324

Rank-biserial correlation coefficient.

**Table 9:** Effect sizes depending on age and BMI vs. VAS.

Kruskal–Wallis test				
Age	**χ^2^**	**df**	***p***	**ε^2^**
VAS	23.8	2	<0.001	0.0122
Kruskal–Wallis test				
BMI	**χ^2^**	**df**	***p***	**ε^2^**
VAS	2.00	3	0.573	0.00103

**Table 10:** Effect sizes depending on age and BMI vs. the need to abandon the procedure.

	Age			BMI		
	Chi-Square	df	*p*	Chi-Square	df	*p*
Chi-square. Pearson	2.197436	df = 2	*p* = 0.33330	2.224216	df = 3	*p* = 0.52719
Chi^2^	2.128129	df = 2	*p* = 0.34505	3.570769	df = 3	*p* = 0.31170
Fi	0.0335692			0.0338339		
Contingency coefficient	0.0335503			0.0338145		
Cramér’s V	0.0335692			0.0338339		

**Table 11:** Effect size depending on the type of hysteroscopy vs. the need to abandon the procedure.

	Chi-Square	df	*p*
Chi-square. Pearson	14.93160	df = 1	*p* = 0.00011
Chi^2^	14.94300	df = 1	*p* = 0.00011
Chi^2^ Yates	13.77735	df = 1	*p* = 0.00021
for 2 × 2 tables	0.0876180		
Tetrachoric correlations	0.3008888		
Contingency coefficient	0.0872836		
Odds ratio and 95.00% CI	3.362774	1.753731	6.448110

**Table 12:** Incidence of early (perioperative) complications—uterine perforation.

Patient 1	The cervical canal was dilated with Hegar dilators up to size 7, followed by insertion of the hysteroscope. A perforation of the uterine cavity at the fundus was observed. Because signs of heavy bleeding were absent, electrosurgical resection of the polyp was performed.
Patient 2	The cervical canal was dilated with Hegar dilators, followed by insertion of the hysteroscope. A uterine perforation was subsequently observed in the left cornu without signs of bleeding.
Patient 3	The uterine cavity was obliterated with the placement of an intrauterine device for approximately 30 years. During the release of adhesions within the uterine cavity, the patient began to experience pain. An iatrogenic uterine perforation in the retroperitoneal space was diagnosed.
Patient 4	A narrow uterine cavity in direct continuity with the cervical canal was observed, with an adhesion at the fundus mimicking an obliterated internal os. During an attempt to dissect the adhesion, the integrity of the uterine muscle was breached, resulting in a defect approximately 5 mm in diameter, without active bleeding.
Patient 5	The uterine cavity and cervical canal were obliterated post endometrial thermoablation. The uterine cavity was completely obliterated. During the release of intrauterine adhesions, a perforation of the uterine wall occurred. The procedure was discontinued without signs of active bleeding into the peritoneal cavity.
Patient 6	Because the cervical canal was obliterated, cervical dilation with Hegar dilators was performed. After the insertion of the hysteroscope, a uterine perforation was detected in the left horn without signs of bleeding.

**Table 13:** Late complications requiring rehospitalization and treatment.

1	Pelvic abscess with peritonitis	Two days after operative hysteroscopy (endometrial polyp resection), the patient was readmitted due to suspected pelvic inflammation and was hospitalized for 8 days. The patient was diagnosed with pelvic abscess and peritonitis. Laparotomy with the removal of adnexa was performed. Antibiotic therapy was administered.
2	Left fallopian tube hydrops	One day after operative hysteroscopy (endometrial polyp resection), the patient was readmitted with suspected left fallopian tube hydrops. Conservative treatment and antibiotics were applied.
3	Acute adnexitis	Six days after operative hysteroscopy (endometrial polyp resection), the patient was readmitted due to lower abdominal pain and was diagnosed with acute adnexitis. Conservative treatment and antibiotics were applied.

## Data Availability

The data presented in this study are available on request from the corresponding author.
